# Maltine

**Published:** 1878-12

**Authors:** 


*British Medical Journal. October* 19, 1878.

"At late meeting of the British Medical Association, at Bath, in August last, among the exhibits of pharmaceutical and medical preparations, much interest was shown in one called *Maltine*, which may be described as a highly concentrated extract of Malted Barley, Wheat and Oats.

"Extracts of Malt (*i. e.* malted barley) are pretty widely known, but this is the first example of a combination of the nutritious principles of these three cereals that we have seen; and the greater value of this combination is apparent, as wheat and oats are especially rich in muscular and fat producing elements. This preparation is entirely free from the products of fermentation, such as alcohol and carbonic acid, and is very agreeable to the taste. Clinical experience enables us to recommend it as a nutritive and digestive agent, in virtue of its albuminoid contents, and its richness in phosphates and in diastase, likely to prove an important remedy in pulmonary affections, debility, many forms of indigestion, imperfect nutrition, and deficient lactation. It will in many cases take the place of Cod-liver oil and pancreatic emulsions, where these are not readily accepted by the stomach.

"The manufacturers, Messrs. Reed & Carnrick. issue a pamphlet describing fully the process of manufacture, which no doubt they will supply to any medical man, and we are disposed to believe that Maltine, which is less known here than abroad, is well worthy of practical attention."

